# Supplementary material for: The Role of Faith-Based Organizations in Improving Vaccination Confidence & Addressing Vaccination Disparities to Help Improve Vaccine Uptake: A Systematic Review
Source: Vaccines (Basel). 2023 Feb 15;11(2):449. doi: 10.3390/vaccines11020449 (PMC9966262; doi:10.3390/vaccines11020449)
Supplement: Supplementary file 1 [file vaccines-11-00449-s001.zip › vaccines-2174324-supplementary.pdf]

Supplementary Material

Supplementary Figure S1: PRISMA Diagram Showing Selection of Articles.

The literature search included an electronic search of articles about FBO programs that supported public health emergency efforts from January 1, 2002 to December 31, 2021. The literature databases searched consisted of PUBMED, Web of Science, Science Direct, and COCHRANE. The search produced a total of 86,891 articles, of which, 76 were removed due to duplicate records, 46,792 were removed for article type, and 25,851 articles were removed for publication year.

The titles and abstracts of 14,172 publications were independently screened on the basis of relevance to topic, resulting in 13,957 publications to be excluded and 215 publications being retrieved for full-text review to further assess eligibility. Of those 215, only 209 were able to be retrieved. These 209 full-text publications were independently screened by 2 reviewers, who assessed eligibility by applying the I/E criteria; 9 of these publications were excluded based on being stand-alone abstracts; 7 were excluded based on being a unpublished article; 12 were excluded based on the Study Quality Assessment Tools developed by the National Heart, Lung, and Blood Institute (NHLBI); 144 were excluded based on the criteria by the Oxford Centre for Evidence-Based Medicine.

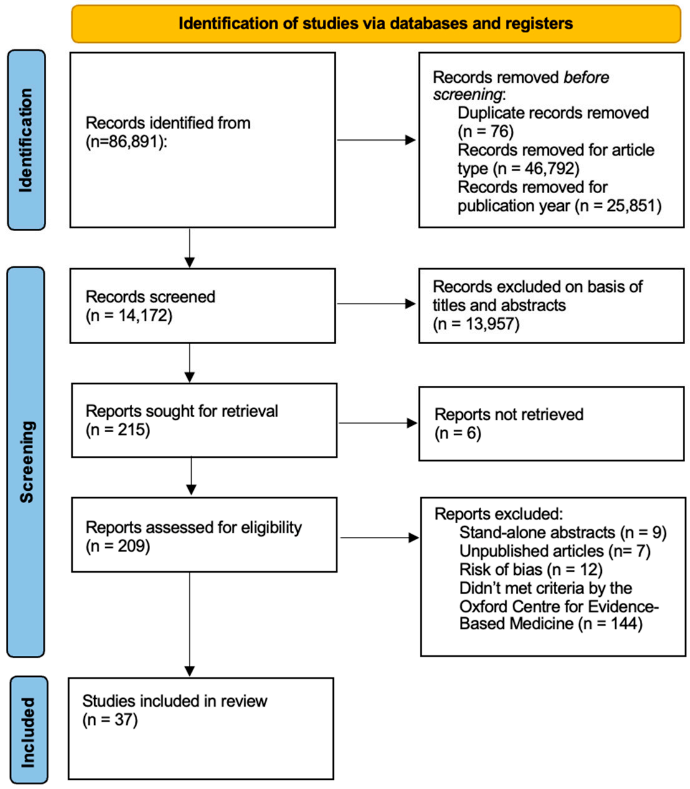

Supplementary Table S1: Sample of quality assessment tool for systematic reviews and meta-analyses by the National Heart, Lung, and Blood Institute (NHLBI) [19] for Sheikhi et al., [7].

Criteria	Yes	No	Other (CD, NR, NA)*
1. Is the review based on a focused question that is adequately formulated and described?	√		
2. Were eligibility criteria for included and excluded studies predefined and specified?	√		
3. Did the literature search strategy use a comprehensive, systematic approach?	√		
4. Were titles, abstracts, and full-text articles dually and independently reviewed for inclusion and exclusion to minimize bias?	√		
5. Was the quality of each included study rated independently by two or more reviewers using a standard method to appraise its internal validity?	√		
6. Were the included studies listed along with important characteristics and results of each study?	√		
7. Was publication bias assessed?		√	
8. Was heterogeneity assessed? (This question applies only to meta-analyses.)			√

*CD, cannot determine; NA, not applicable; NR, not reported.

Supplementary Table S2: Sample of quality assessment tool for observational cohort and cross-sectional studies by the National Heart, Lung, and Blood Institute (NHLBI) [19] for Olagoke et al., [50].

Criteria	Yes	No	Other (CD, NR, NA)*
1. Was the research question or objective in this paper clearly stated?	√		
2. Was the study population clearly specified and defined?	√		
3. Was the participation rate of eligible persons at least 50%?			√
4. Were all the subjects selected or recruited from the same or similar populations (including the same time period)? Were inclusion and exclusion criteria for being in the study prespecified and applied uniformly to all participants?	√		
5. Was a sample size justification, power description, or variance and effect estimates provided?			√
6. For the analyses in this paper, were the exposure(s) of interest measured prior to the outcome(s) being measured?		√	
7. Was the timeframe sufficient so that one could reasonably expect to see an association between exposure and outcome if it existed?			√
8. For exposures that can vary in amount or level, did the study examine different levels of the exposure as related to the outcome (e.g., categories of exposure, or exposure measured as continuous variable)?			√
9. Were the exposure measures (independent variables) clearly defined, valid, reliable, and implemented consistently across all study participants?	√		
10. Was the exposure(s) assessed more than once over time?		√	
11. Were the outcome measures (dependent variables) clearly defined, valid, reliable, and implemented consistently across all study participants?	√		
12. Were the outcome assessors blinded to the exposure status of participants?			√
13. Was loss to follow-up after baseline 20% or less?			√
14. Were key potential confounding variables measured and adjusted statistically for their impact on the relationship between exposure(s) and outcome(s)?	√		

*CD, cannot determine; NA, not applicable; NR, not reported.

Supplementary Table S3: Sample of quality assessment tool for controlled intervention studies by the National Heart, Lung, and Blood Institute (NHLBI) [19] for Daniels et al., [51].

Criteria	Yes	No	Other (CD, NR, NA)*
1. Was the study described as randomized, a randomized trial, a randomized clinical trial, or an RCT?	√		
2. Was the method of randomization adequate (i.e., use of randomly generated assignment)?	√		
3. Was the treatment allocation concealed (so that assignments could not be predicted)?	√		
4. Were study participants and providers blinded to treatment group assignment?	√		
5. Were the people assessing the outcomes blinded to the participants' group assignments?		√	
6. Were the groups similar at baseline on important characteristics that could affect outcomes (e.g., demographics, risk factors, co-morbid conditions)?	√		
7. Was the overall drop-out rate from the study at endpoint 20% or lower of the number allocated to treatment?			√
8. Was the differential drop-out rate (between treatment groups) at endpoint 15 percentage points or lower?			√
9. Was there high adherence to the intervention protocols for each treatment group?	√		
10. Were other interventions avoided or similar in the groups (e.g., similar background treatments)?	√		
11. Were outcomes assessed using valid and reliable measures, implemented consistently across all study participants?	√		
12. Did the authors report that the sample size was sufficiently large to be able to detect a difference in the main outcome between groups with at least 80% power?		√	
13. Were outcomes reported or subgroups analyzed prespecified (i.e., identified before analyses were conducted)?	√		
14. Were all randomized participants analyzed in the group to which they were originally assigned, i.e., did they use an intention-to-treat analysis?	√		

*CD, cannot determine; NA, not applicable; NR, not reported.

Supplementary Table S4: Sample of quality assessment tool for case series studies by the National Heart, Lung, and Blood Institute (NHLBI) [19] for Dascalu et al., [53].

Criteria	Yes	No	Other<br>(CD, NR, NA)*
1. Was the study question or objective clearly stated?	√		
2. Was the study population clearly and fully described, including a case definition?	√		
3. Were the cases consecutive?	√		
4. Were the subjects comparable?	√		
5. Was the intervention clearly described?	√		
6. Were the outcome measures clearly defined, valid, reliable, and implemented consistently across all study participants?	√		
7. Was the length of follow-up adequate?	√		
8. Were the statistical methods well-described?			√
9. Were the results well-described?	√		

*CD, cannot determine; NA, not applicable; NR, not reported.
